# Breastfeeding vs. formula feeding on maternal oral health: periodontal and microbiological changes in the postpartum period: an observational longitudinal study

**DOI:** 10.3389/froh.2026.1747704

**Published:** 2026-03-12

**Authors:** Federica Macrì, Gianna Dipalma, Angelo Michele Inchingolo, Francesco Inchingolo, Cinzia Maspero, Lucia Giannini

**Affiliations:** 1 Department of Biomedical, Surgical and Dental Sciences, University of Milan, Milan, Italy; 2 Fondazione IRCCS Cà Granda, Ospedale Maggiore Policlinico, Milan, Italy; 3 Department of Life Science, Health and Health Professional, Link Campus University, Roma, Italy; 4 Department of Interdisciplinary Medicine, University of Bari Aldo Moro, Bari, Italy

**Keywords:** breastfeeding, infant nutrition, maternal oral health, microbiota, periodontal indices, postpartum period, streptococcus mutans

## Abstract

**Introduction:**

Breastfeeding is widely recognized for its benefits to infant health, yet its potential effects on maternal oral health are rarely addressed.

**Methods:**

This observational longitudinal study evaluated 90 postpartum period mothers, divided into breastfeeding (*n* = 55) and formula-feeding (*n* = 35) groups, assessing periodontal and microbiological parameters at baseline postpartum (T0), 15 ± 2 days (T1), 40 ± 3 days (T2), and six months postpartum (T3). All participants received standardized oral hygiene education.

**Results:**

While both groups improved initially, formula-feeding mothers showed progressive improvement through 6 months. In breastfeeding mothers, GBI (bleeding index) increased again at T3 vs. T2, whereas PI (plaque index) returned to T1 levels and *S. mutans* and *Lactobacilli* decreased by T3; however, at T3 the BF (breastfeeding) group still showed higher PI/GBI and cariogenic bacteria than FF (formula feeding).

**Discussion:**

These results suggest that the cumulative physical and psychological strain associated with breastfeeding may be associated with reduced oral-hygiene adherence, showing an association with maternal oral health. Targeted oral prevention strategies and inclusion of maternal dental monitoring in perinatal care protocols may help mitigate these risks without compromising the recognized benefits of breastfeeding.

## 1 Introduction

A recent systematic review has highlighted how maternal and neonatal oral microbiome developmental patterns are strongly interconnected, with early feeding practices shaping microbial colonization from the very first months of life (1). This evidence reinforces the concept that infant oral health cannot be considered in isolation, but is deeply influenced by maternal health conditions and feeding choices.

Pregnancy represents a crucial period influencing both maternal and infant future health.

Recent research has shown that the maternal microbiota, especially the oral and gut microbiomes, undergoes dynamic changes during pregnancy and plays a key role in early microbial seeding of the newborn, influencing baby's immune system maturation and susceptibility to future diseases (1, 2).

Maintaining maternal oral and systemic health during pregnancy is essential, as maternal dysbiosis can affect not only obstetric outcomes but also the establishment of the infant's microbiota after birth.

This underscores the importance of maternal consciousness and preventive health behaviors even before delivery; mothers must be educated to start perinatal care during pregnancy rather than postpartum.

Furthermore, studies conducted in Spain and France revealed that oral health knowledge, attitudes, and practices among breastfeeding mothers are still inadequate, suggesting that maternal awareness is a critical determinant of both maternal and infant oral outcomes (3).

Beyond infant outcomes, breastfeeding exerts significant effects on maternal health. The peripartum period is characterized by profound hormonal and metabolic changes that may alter salivary composition and influence periodontal status (4, 5). Physiological demands of lactation can affect metabolic and cardiovascular markers, while psychosocial stressors—including sleep deprivation, co-sleeping, and maternal mental health challenges—have been shown to compromise adherence to preventive practices and oral self-care (6–9).

In this context, several authors have emphasized that breastfeeding may also impact maternal oral health. Reviews have highlighted systemic and oral health benefits of breastfeeding (10), but methodological challenges remain (11). Earlier contributions recognized maternal oral health as a critical component of postpartum care, calling for greater involvement of dental practitioners (12, 13).

Additionally, maternal oral dysbiosis during the postpartum period, especially in breastfeeding mothers, has been linked to increased colonization by cariogenic species such as Streptococcus mutans and Lactobacilli (14–18).

Recent investigations further support this association: maternal oral health has been linked to obstetric risk and breastfeeding practices (19); breastfeeding has been associated with an increased prevalence of periodontitis in Korean women (20); and the duration of breastfeeding may even influence oral health later in life (21).

Crucially, maternal systemic health is not only crucial for the mother herself but also for the child. This highlights how maternal-focused interventions may have intergenerational benefits, extending beyond general outcomes to include oral and dental health.

Despite growing evidence on the interplay between infant feeding and oral health, the impact of breastfeeding on maternal oral conditions during the postpartum period remains poorly investigated.

The aim of the present longitudinal observational study was to assess changes in maternal periodontal and microbiological parameters during the postpartum period, by comparing breastfeeding and formula-feeding mothers.

We hypothesized that the cumulative physical and psychological demands of breastfeeding may influence maternal oral health outcomes, underscoring the need for preventive strategies tailored to maternal needs.

## 2 Materials and methods

This observational longitudinal study included a total of 90 mothers, divided into two groups based on their infant feeding method: 55 breastfeeding mothers (BF) and 35 formula-feeding mothers (FF). Mothers were consecutively recruited at the maternity wards of Fondazione IRCCS Cà Granda, Ospedale Maggiore Policlinico (Milan) and the University Hospital of Bari. After enrollment, participants were followed up in the dental clinics of the same institutions, where they underwent oral examinations and microbiological sampling.

The study was approved by the Ethics Committee (D.G.R. Regione Puglia n.712 del 22/05/2023 e rett. N. 806 del 12/06/2023). All mothers were fully informed about the study procedures, both orally and in writing, and provided written consent prior to participation, in accordance with the Declaration of Helsinki.

### 2.1 Selection criteria

Participants were selected according to predefined inclusion and exclusion criteria, with the goal of minimizing potential confounding factors and ensuring greater homogeneity between the study groups.

Inclusion Criteria:

1. Mothers aged between 20 and 45 years.
2. Had Recently given natural birth to a single child, with no preventive dental care during pregnancy
3. Willing to exclusively breastfeed or exclusively formula-feed their infant for at least 6 months.
4. Good general health, without systemic conditions that may influence oral health (except for temporary postpartum period hormonal changes).
5. Availability to attend all follow-up visits (T1, T2, T3).
6. Provided signed informed consent to participate in the study.

Exclusion Criteria:

1. Presence of systemic diseases or conditions known to affect periodontal health (e.g., diabetes, immunodeficiencies).
2. Use of antibiotics or antimicrobial mouth rinses in the 30 days prior to or during the study period.
3. Current smokers or former smokers who quit less than 6 months prior.
4. Mothers undergoing orthodontic treatment or with extensive prosthetic rehabilitation.
5. Mothers with known allergies to components of the microbiological transport media.
6. Poor compliance with the study protocol or missing any of the follow-up evaluations.
7. Mothers who did not deliver naturally and who did not exclusively breastfeed or formula-feed

All participants were enrolled following a standardized preventive dental care protocol referred to as the “preventive iter”. This protocol emphasizes individualized oral hygiene education, regular monitoring, and professional dental care to manage and prevent oral health complications associated with pre and postpartum period. The Preventive Iter is divided into four stages, in which specific and standardized procedures are performed on patients; subsequently checkups are planned.

The “Preventive Iter” protocol, originally developed for perinatal populations including pregnancy, was adapted to the postpartum setting in this study. It was initiated at T0, the first postpartum visit (usually 48 h after hospital discharge, which usually occurs 2 days after birth) and reinforced at each subsequent visit.

A total of 118 mothers were screened during the recruitment period (January 2023–January 2024). Of these, 28 were excluded because they did not meet the inclusion criteria or declined participation. Ninety mothers (participation rate: 76%) were finally enrolled, of whom 55 were breastfeeding and 35 formula-feeding.

Exclusive breastfeeding or formula feeding status was verified at each follow-up visit through structured interviews. If any mother had introduced formula or complementary feeding before 6 months, she would have been excluded from the breastfeeding group.

### 2.2 Potential confunders

Potential confounders were extracted from institutional obstetric records and the integrated clinical database, since all participants were followed within an obstetric unit formally connected to our Department. Extracted variables included self-reported ethnicity and occupation status (routine obstetric intake), dietary profile (varied vs. restrictive patterns; vegetarian/vegan status), daily frequency of sugary meals/snacks, and baseline caries experience measured as DMFT, PI and GBI at the first-trimester dental visit. These variables were used to characterize baseline comparability between groups (Supplementary Table S1) and to contextualize the interpretation of postpartum oral-hygiene and gingival-inflammation trajectories.

Ethnicity was self-reported at routine obstetric intake. For reporting purposes, categories were harmonized using contemporary descriptors: White/Caucasian, Asian, Black/African, Oceanian, Southeast Asian, and Other/Mixed. When small cell counts occurred, categories were retained for transparency and group comparisons were interpreted cautiously.

### 2.3 Follow-Up assessment

Assessments were performed at four time points: T0 (baseline), T1(15 ± 2 days postpartum), T2 (40 ± 3 days postpartum), and T3 (6 months postpartum).

All visits were scheduled in advance, and reminders were sent by phone and text message 48 h beforehand to minimize missed appointments.

The following clinical and microbiological variables were recorded at each time point:

- Plaque Index (PI)
- Gingival Bleeding Index (GBI)
- Total cocci and bacilli count
- Gram-positive and Gram-negative cocci and bacilli
- Streptococcus mutans count
- Lactobacilli count

Periodontal indices were evaluated by a calibrated examiner using standardized clinical methods to ensure consistency and reliability of measurements. To improve measurement reliability, all clinical evaluations were performed by a single calibrated examiner, while microbiological samples were collected by trained staff under standardized aseptic conditions. At each visit, adherence to the preventive protocol (Preventive Iter) was reinforced through brief motivational counseling and oral hygiene instruction. Microbial samples were collected from the buccal and interproximal areas using sterile swabs and were transported in appropriate media for assessment. Microbiological assessment was conducted through selective culture techniques and Gram staining. Identification and quantitation of S. mutans and Lactobacilli was carried out by culture on selective agar plates (Mitis Salivarius for S. mutans, Rogosa SL agar for Lactobacilli) and subsequent counting of the colonies under standardized conditions.

All subjects received standardized oral hygiene instructions (correct toothbrushing technique and use of interdental aids) at T0. Professional prophylaxis was not performed during the study in order to avoid external influence on the outcome.

### 2.4 Sample size and statistical analysis

The main outcome of interest was the change in maternal periodontal health over time, evaluated through the Plaque Index (PI) and Gingival Bleeding Index (GBI). Secondary outcomes included changes in microbial composition, namely total cocci and bacilli, Gram-positive and Gram-negative subgroups, as well as Streptococcus mutans and Lactobacilli.

An *a priori* power analysis was conducted for a repeated-measures ANOVA focusing on the group-by-time interaction across four time points. Assuming a medium effect size (f = 0.25), an alpha level of 0.05, and a power of 0.80 (based on previous prevention studies in perinatal contexts) this was considered to represent a clinically meaningful moderate difference in periodontal indices and cariogenic bacterial trajectories between groups. Under these parameters, the required minimum sample size was 76 participants. To account for an estimated 15% dropout rate, 90 mothers were enrolled (55 breastfeeding and 35 formula-feeding).

Because statistical power in repeated-measures designs depends on within-subject correlation (*ρ*) and potential non-sphericity adjustments (*ε*), a sensitivity analysis was also performed for the group-by-time interaction. With *ρ* = 0.50 and *ε* = 1.00, the achieved power was approximately 87%. Testing a range of plausible values (*ρ* = 0.30–0.70; *ε* = 0.90–1.00) yielded an estimated power between 83% and 89%, comfortably above the conventional 80% threshold (STATA Software).

Data are presented as mean ± SD. The Shapiro–Wilk test was used to assess the normality of data distribution. Since all variables showed a normal distribution, parametric statistics were applied. Changes within each group across timepoints (T0, T1, T2, T3) were analyzed using repeated-measures ANOVA, with the Greenhouse–Geisser correction applied when the sphericity assumption was violated. When overall effects were significant, pairwise comparisons were conducted using Student's *t*-tests—paired *t*-tests for within-group changes and independent *t*-tests for between-group differences—applying Bonferroni adjustment for multiple testing. Effect sizes were calculated as Cohen's d for pairwise comparisons and partial eta-squared (*η*^2^) for ANOVA models. Statistical significance was set at *α* = 0.05. Missing data were handled by listwise deletion; however, no imputation was required as all participants completed follow-up. These procedures were adopted to enhance the robustness of the findings despite the exploratory nature of the study.

All analyses were performed using IBM SPSS Statistics for Windows, Version 29.0 (IBM Corp., Armonk, NY, USA).

## 3 Results

Baseline demographic, socioeconomic and dietary characteristics (including ethnicity, occupation status, dietary profile, sugary-meal frequency, and first-trimester DMFT) were comparable between breastfeeding and formula-feeding mothers (Table 1; Supplementary Table S1).

**Table 1:** Mean values and standard deviations of periodontal and microbiological parameters in breastfeeding mothers at T0, T1, T2 and T3. PI = plaque index, GBI, gingival bleeding index; G+, gram positive; G-, gram negative.

PI				COCCI TOT			
T0	T1	T2	T3	T0	T1	T2	T3
0,49	0,19	0,24	0,19	232,4	75,18	86,4	86,53
0,14	0,08	0,09	0,06	107,74	41,2	48,49	32,04
BACILLI TOT				COCCI G+			
T0	T1	T2	T3	T0	T1	T2	T3
71,15	23,75	33,18	26,04	2,76	1,44	1,91	1,18
49,06	16,65	21,44	16,09	0,51	0,57	0,4	0,39
COCCI G-				BACILLI G+			
T0	T1	T2	T3	T0	T1	T2	T3
1,95	0,8	1,2	0,85	1,89	1,02	1,42	1
0,8	0,52	0,49	0,59	0,63	0,3	0,53	0,27
BACILLI G-				S. MUTANS			
T0	T1	T2	T3	T0	T1	T2	T3
1,44	0,53	0,62	0,61	30,16	17,11	12,31	8,45
0,57	0,57	0,59	0,5	12,63	7,19	10,34	1,37
LACTOBACILLI				GBI			
T0	T1	T2	T3	T0	T1	T2	T3
22,53	12,24	8,91	8,04	2,2	1,09	0,76	1,33
9,4	4,01	1,16	1,84	2,68	0,67	1,49	0,51

Both groups at T0 (baseline) had equivalent plaque index (PI), gingival bleeding index (GBI), and microbiological values and lacked statistically significant differences (*p* > 0.05) (Table 1; Table 2; Supplementary Tables S2–S4).

**Table 2:** Mean values and standard deviations of periodontal and microbiological parameters in formula-feeding mothers at T0, T1, T2, and T3. PI, plaque index; GBI, gingival bleeding index; G+, gram positive; G-, gram negative.

PI				COCCI TOT			
T0	T1	T2	T3	T0	T1	T2	T3
0,43	0,2	0,14	0,07	218	72,53	68,66	42,6
0,16	0,08	0,09	0,05	115,05	35,87	43,9	30,76
BACILLI TOT				COCCI G+			
T0	T1	T2	T3	T0	T1	T2	T3
65,54	20,63	17,23	7,9	2,57	1,46	1,87	1,13
29,94	12,86	12,95	6,57	0,67	0,55	0,51	0,37
COCCI G-				BACILLI G+			
T0	T1	T2	T3	T0	T1	T2	T3
2,01	0,79	1,11	0,64	1,77	0,97	1,3	0,96
0,85	0,53	0,49	0,59	0,72	0,39	0,55	0,34
BACILLI G-				S. MUTANS			
T0	T1	T2	T3	T0	T1	T2	T3
1,38	0,44	0,54	0,24	38,78	16,37	10,01	6,91
0,57	0,53	0,49	0,42	16,33	9,36	5,53	2,25
LACTOBACILLI				GBI			
T0	T1	T2	T3	T0	T1	T2	T3
27,36	9,15	7,89	5,24	1,4	0,7	0,5	0,14
14,84	3,31	2,69	1,44	0,92	0,2	0,1	0,14

Following oral hygiene instructions provided at T0, both groups showed overall improvement in clinical and microbiological values at T1. This was evidenced by a reduction in plaque index (PI), gingival bleeding index (GBI), and number of bacteria, particularly *S. mutans* and *Lactobacilli* (Table 1; Table 2; Supplementary Tables S2–S4).

**Figure 1:**
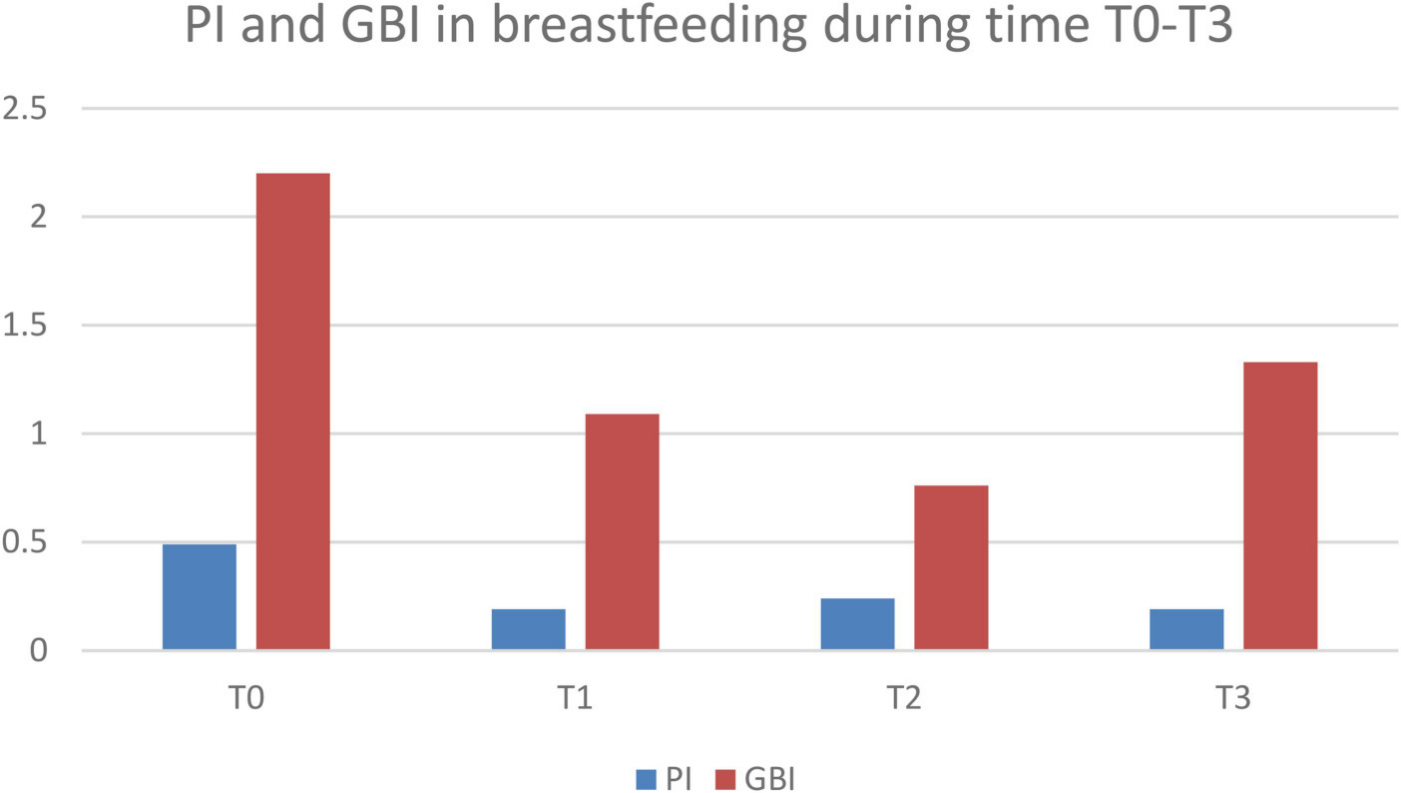
Plaque Index (PI) and gingival bleeding index (GBI) in breastfeeding mothers (BF) at timepoints T0–T3. Data are presented as mean ± SD.

However, there were diverging trends in the two groups across time. In breastfeeding mothers clinical and microbial parameters improved at T1 but did not continue to decrease thereafter. Specifically, after initial improvement at T1, parameters in breastfeeding mothers remained stable until T2, followed by a significant rebound at T3. From T1 to T3, breastfeeding (BF) mothers showed stable PI (with PI at T3 comparable to T1), an increase in GBI at T3 vs. T2, and a decrease in *S. mutans* and *Lactobacilli* counts at T3 (Table 1; Supplementary Tables S2 and S3; Figure 1).

**Figure 2:**
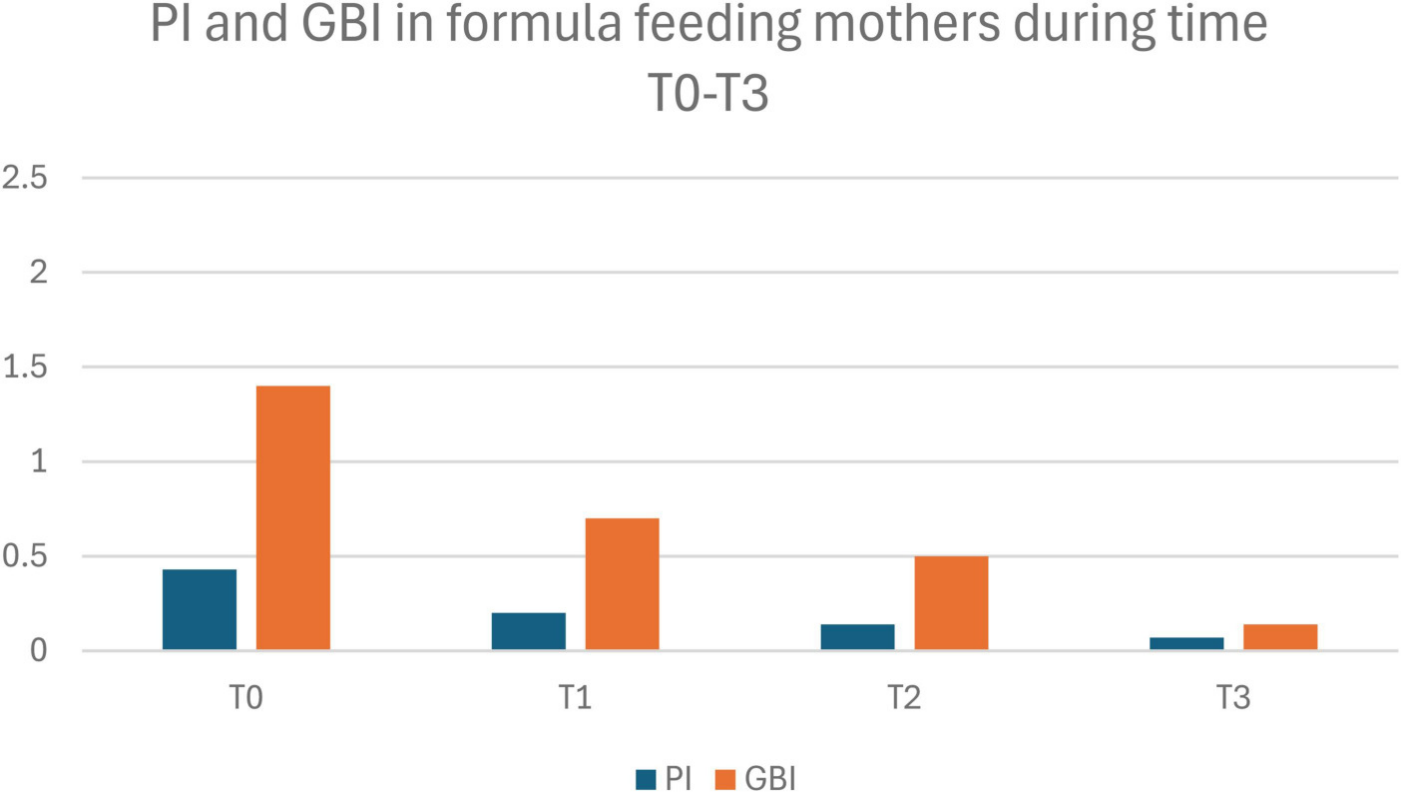
Plaque Index (PI) and gingival bleeding index (GBI) in formula-feeding mothers (FF) at timepoints T0–T3. Data are presented as mean ± SD.

In contrast, the formula-fed mothers experienced a more controlled environment over the study period. While they also improved initially at T1, periodontal and microbiological parameters showed progressive improvement throughout the study. In fact, at T3, the formula-fed (FF) Group showed significantly lower values for plaque index (PI), bleeding, total bacterial count, and cariogenic microorganisms compared to the BF group (*p* < 0.05). At T3, BF mothers showed higher cariogenic bacterial counts than FF (Table 2; Supplementary Tables S2–S4; Figure 2).

Table 2 summarizes the between-group comparisons for PI and GBI with no relevant differences at baseline (T0). However, from T1 onward breastfeeding mothers consistently showed higher PI values compared with formula-feeding mothers, with the divergence becoming significant at T3. Similarly, GBI values were higher in breastfeeding mothers across follow-ups, differences became evident from T2 and were more pronounced at T3 (Supplementary Table S2).

Figures 1, 2 illustrate within-group changes in PI and GBI across time points for breastfeeding and formula-feeding mothers, respectively. The graphs clearly illustrate the different trends over time (Figures 1, 2).

The overall effect of time and group, tested with ANOVA models, was statistically significant (*p* < 0.05). Significant main effects of time and group, and a significant time × group interaction were found for both indices (*p* < 0.05).

Repeated-measures analysis showed significant main effects of time and a significant time  ×  group interaction for both PI and GBI (*p* < 0.05), indicating divergent postpartum trajectories between BF and FF mothers. While both groups improved after standardized oral-hygiene counseling (T1), FF mothers showed progressive improvement through T3, whereas BF mothers showed stabilization and a late rebound in gingival bleeding. The largest between-group differences were observed at T3 (Supplementary Tables S2–S4; Figures 1, 2), with corresponding effect sizes reported to aid clinical interpretation.

## 4 Discussion

In this longitudinal cohort of 90 postpartum mothers managed under a standardized preventive protocol, both breastfeeding (BF) and formula-feeding (FF) groups improved shortly after baseline; however, their trajectories diverged thereafter. FF mothers showed continued improvement through 6 months, whereas BF mothers experienced stabilization and, for gingival bleeding, a late rebound, with higher PI/GBI and greater cariogenic bacterial burden at T3 than FF (*p* < 0.01 for several comparisons after multiplicity adjustment where applicable). These patterns were observed despite uniform counseling and follow-up, suggesting that infant feeding modality is associated with distinct maternal oral-health trajectories in the early postpartum period. Importantly, these findings should not be interpreted as indicating a detrimental effect of breastfeeding itself on maternal oral health or a causal factor in the observed oral health changes; rather, the associations identified are more likely to reflect the complex physical, hormonal, and organizational demands of the postpartum period, which may differentially affect maternal self-care behaviors.

From a clinical perspective, the observed time  ×  group interaction suggests that breastfeeding mothers may experience greater difficulty in maintaining long-term plaque control and gingival stability during the postpartum period, despite initial improvement. Importantly, these differences are not merely statistically significant but also clinically relevant, supporting the need for reinforced preventive strategies in this population.

Our findings align with prior studies linking breastfeeding to specific maternal oral health associations. Serrano-Sánchez et al. (19) identified an association between maternal oral status, obstetric complications and breastfeeding, while Wang (20) demonstrated a higher prevalence of periodontitis among breastfeeding women in Korea. Furthermore, Buali and Delgado-Angulo (21) suggested that breastfeeding duration may have long-term implications for oral health, reinforcing the need to monitor maternal conditions beyond the immediate postpartum period.

At the same time, our results nuance the common assumption that breastfeeding confers universal benefits across all domains: while the infant benefits are well established, mothers in the BF group may require additional support to sustain oral-hygiene behaviors, plaque control and gingival inflammation (10–13). From a public health perspective, these findings underscore the need for enhanced maternal support during breastfeeding rather than questioning the value of breastfeeding itself.

Interestingly, we found that mothers who formula-fed their babies showed steady improvements in both clinical and microbiological aspects, which aligns with existing research that connects more regular daily routines to better self-care and preventive practices.

While breast milk is known for its antibacterial properties largely attributed to lactoperoxidase, immunoglobulin A, and oligosaccharides (18), the local benefits on oral health appear to be more relevant for the infant rather than the mother. Several studies report that the composition of mothers' saliva during breastfeeding may be altered in its antimicrobial components in response to hormonal and metabolic variations, resulting in changes in the maternal oral biofilm (4, 5).

Postpartum biological and behavioural pathways may jointly underlie the BF–FF divergence. Hormone-driven changes can affect salivary composition and gingival health, while fragmented sleep and caregiving demands may affect daily hygiene routines. Although maternal psychological status was not assessed here, prospective studies link breastfeeding with distinct postpartum mood trajectories and differing risks of depression compared with non-breastfeeding women; maternal mental health may therefore mediate adherence to oral-hygiene and preventive behaviours and warrants explicit evaluation in future work (9, 22–26).

Beyond its nutritional and immunological benefits, breastfeeding plays a critical role in the proper development of the infant's orofacial structures. The physiological sucking required during breastfeeding stimulates a balanced activity of perioral, lingual, and masticatory muscles, helping mandibular advancement and functional growth of the oral cavity. This important muscular coordination supports normal swallowing and physiological breathing patterns, reducing the risk of developing malocclusions later in childhood.

Moreover, breastfeeding helps mother–infant bonding through close physical and emotional contact, which is important for their future development.

Breastfeeding also shapes both nutritive and non-nutritive sucking habits, helping to prevent the persistence of deleterious oral habits such as pacifier or thumb sucking that can alter dental arch development (5, 27). These early functional benefits have lifelong implications, as they influence craniofacial growth, airway development and overall oral health.

Therefore, the balance should be sought not in the replacement or discouragement of breastfeeding, but in the integration of targeted maternal support, recognizing the practical difficulties this choice entails and providing personalized follow-up for maternal oral health (28–32).

In this context, the role of healthcare providers appears central. As highlighted, the inclusion of maternal oral hygiene in perinatal protocols could represent an effective strategy to counteract the oral health deterioration observed in breastfeeding mothers (6, 7). Concurrently, educational programs directed at partners and extended family have proven useful in supporting mothers in maintaining personal care (33–39).

### 4.1 Strengths and limitations of the study

Key strengths of this study include the prospective design with repeated, standardized assessments at multiple postpartum time points; the combined evaluation of clinical and microbiological outcomes; and the application of a uniform preventive protocol across groups, which limits variability in counseling and follow-up.

This study has some limitations. First, the sample size was modest and the follow-up was limited to six months, which constrains the robustness and generalizability of the findings; extending beyond six months was not feasible because complementary feeding typically begins thereafter. Second, maternal psychological status assessed with validated instruments. Infant clinical parameters together with baseline family caries experience and household economic circumstances were not recorded. Finally, mothers' education/occupation were not included as covariates in the primary models and socioeconomic confounding remains a concern. These unmeasured psychosocial and contextual factors are highly relevant in the postpartum period and may partially or fully account for the differences observed between breastfeeding and formula-feeding mothers.

Although true preconception oral-health measures were not available, first-trimester DMFT, PI and GBI provided an objective proxy of baseline caries experience prior to the postpartum follow-up.

Future studies should enroll larger cohorts, incorporate validated psychosocial and socioeconomic measures, and employ advanced microbiological approaches (16S rRNA sequencing) in order to better characterize postpartum oral-biofilm dynamics.

### 4.2 Implications for clinical practice

As PI and GBI are well-established markers to monitor oral hygiene and gingival inflammatory status, but do not allow a diagnosis of periodontitis, the conclusions of this study are intentionally limited to hygiene- and inflammation-related outcomes.

Our data indicate that standard counseling may not suffice to sustain oral health in BF mothers over 6 months. Incorporating brief, structured reinforcement at well-baby or lactation visits (technique checks, interdental aid training, tailored reminders) and considering simple adjuncts with demonstrated antimicrobial effects could be pragmatic.

## 5 Conclusions

This longitudinal study demonstrated a significant association between infant feeding modality and the evolution of maternal oral health during the postpartum period. While both groups initially benefited from a standardized oral hygiene education protocol, formula-feeding mothers showed a more stable maintenance of periodontal and microbiological parameters over time, whereas breastfeeding mothers tended to lose part of the initial improvement, with a rebound at 6 months.

These findings suggest that the observed changes in maternal oral health are not directly attributable to breastfeeding itself, but rather to the broader physical and psychological demands of the postpartum period, such as disrupted routines, sleep deprivation, and reduced time for self-care. Importantly, breastfeeding should still be regarded as the optimal form of nutrition for infants, given its well-established benefits for neonatal health and development.

Microbiological analyses confirmed a higher bacterial load in breastfeeding mothers compared to formula-feeding mothers, particularly for Streptococcus mutans and Lactobacilli, indicating changes in oral biofilm composition that may be influenced by hormonal fluctuations and challenges in maintaining optimal daily oral hygiene during the postpartum period.

Nevertheless, it is important to emphasize that breastfeeding remains the gold standard for infant nutrition, given its well-established systemic and developmental benefits.

Despite the study's limitations—including the modest sample size, relatively short follow-up, absence of validated psychological assessments, lack of standardized epidemiological indices (DMFT, orthodontic status), and omission of a structured analysis of socioeconomic factors—our results highlight the importance of integrating maternal oral health monitoring into perinatal care.

Recent evidence (28–30) supports the effectiveness of structured preventive programs, such as the “Preventive Iter” protocol, in reducing bacterial colonization and improving outcomes in systemically vulnerable populations. Translating such strategies into perinatal care could help mitigate oral health risks in breastfeeding mothers without compromising the benefits of breastfeeding itself.

Future studies should involve larger and more diverse cohorts, extend follow-up beyond six months, systematically include psychosocial and socioeconomic assessments, and employ advanced microbiological techniques such as 16S rRNA sequencing. These approaches will be essential to better understand the multifactorial determinants of maternal oral health during breastfeeding and to develop tailored preventive strategies that can be integrated into routine perinatal care.

## Data Availability

The original contributions presented in the study are included in the article/Supplementary Material, further inquiries can be directed to the corresponding authors.
